# Setup and Analysis of a Mid-Infrared Stand-Off System to Detect Traces of Explosives on Fabrics

**DOI:** 10.3390/s22207839

**Published:** 2022-10-15

**Authors:** Lisa B. Dreier, Christoph Kölbl, Vincent Jeuk, Claudia Beleites, Anja Köhntopp, Frank Duschek

**Affiliations:** 1 German Aerospace Center, Institute of Technical Physics, Im Langen Grund 1, 74239 Hardthausen, Germany; 2 Chemometrix GmbH, Södeler Weg 19, 61200 Wölfersheim, Germany

**Keywords:** laser based explosives detection, remote sensing, EC-QCL, selective screening, security checkpoints, automation

## Abstract

The increasing number of terrorist attacks within the last decade has demonstrated that taking preventive protective measures is highly important. In addition to existing measures, automated detection systems for fast and reliable explosive detection are required. A sensitive spectroscopic system based on mid-infrared spectroscopy has been developed and applied to explosive samples on different types of fabric under various geometric conditions. Using this system, traces of TNT, RDX, PETN and ammonium nitrate can be detected in less than a second. Various approaches for data pretreatment (wavelength calibration) and subsequent analysis (normalization, removal of atmospheric water absorption lines) are presented and the remaining challenges on the road to a fully automated system, including a robust classification algorithm, are discussed.

## 1. Introduction

An increasing number of terrorist attacks within the last decade, such as the one in Brussels in 2016, has demonstrated that taking preventive protective measures against such threats is highly important. One key element to improving safety standards is the fast and reliable detection of explosives. At security checkpoints, metal detectors and X-ray spectroscopy are mostly being used [1,2]. Selective screening for explosives is primarily performed by ion mobility spectroscopy in the so-called swab tests or by using canines [3,4]. Optical methods overcome the disadvantages of these resource-intensive technologies as they usually allow for fast, selective and sensitive detection of hazardous substances that have the potential for automated operation. Additionally, the fact that such methods permit contact-free detection is rather intriguing since operating from afar when screening for explosive materials obviously provides an increase in safety for personnel [1]. A recent summary of laser-based optical methods to detect hazardous materials can be found in reference [5]. Infrared (IR) spectroscopy is a promising optical technique that uses light absorption by energetic vibrations of molecules to identify explosives by Fourier-transform techniques or in the frequency domain [6,7,8,9,10,11,12,13,14]. The invention of quantum cascade lasers (QCLs) has particularly enabled the detection of trace materials at distances of up to one hundred meters [14], as QCLs exhibit an increased output power of up to hundreds of milliwatts. Stable operation in a large spectrum of wavelengths, mode-hop-free tuning, and a narrow line width at various operating conditions are other factors that make QCLs interesting for detection of hazardous substances [15,16,17] and gas analysis [18,19].

Here, we present a new laser-based stand-off system for explosives detection on fabrics that has been developed as a featured part of security checkpoint applications. Requirements for this scenario were components that had highly selective detection and the potential for system stability and integration. As most explosive materials exhibit characteristic absorption features in the wavelength range between 6 and 11 μm (around 900–1650 cm^−1^) [20], we used a quantum cascade laser that is tunable in that range. In stand-off geometry, hazardous materials can be detected in a contact-free mode, which was realized in the present investigation by recording the absorption features of back-reflected and back-scattered light intensities. Ideally, the acquired spectra would be analyzed automatically and set off an alarm if hazardous material were detected. A classification algorithm for fast data analysis needs to be trained using a large variety of data that have to be generated by measurements and can be helped by the development of a reliable model to augment the data. Each of these aspects has its disadvantages: An enormous number of measurements is needed, which is laborious and makes taking all possible influencing factors (e.g., types of fabrics, geometries …) into a model with a high enough accuracy impossible [16,21,22]. In a first step toward such automatic detection and classification, the IR spectra of a variety of explosives such as trinitrotoluene (TNT) and royal demolition explosive (RDX) were measured at several angles of incidence, on multiple substrates and using varied amounts of sample material. Furthermore, the spectra of a number of harmless substances were recorded as a reference. The resulting spectra were analyzed, and an approach for data pre-processing prior to classification is discussed in this paper.

## 2. Materials and Methods

### 2.1. Experimental Setup

**Figure 1:**
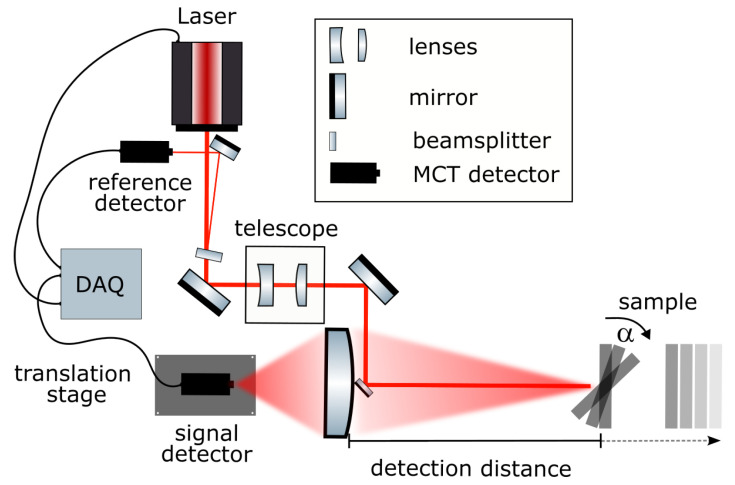
Schematic drawing of the MIR reflection setup.

An overview of the detection system is given in Figure 1. The expanded IR laser beam is guided into a telescope lens for beam shaping and then directed onto the sample by a small outcoupling mirror as a collimated beam with a diameter of 2 mm. Backscattered signals were collected by a planoconvex lens that was collinearly aligned to the excitation beam and detected by a single-point mercury cadmium telluride (MCT) sensor placed on a linear translation stage.

**Figure 2:**
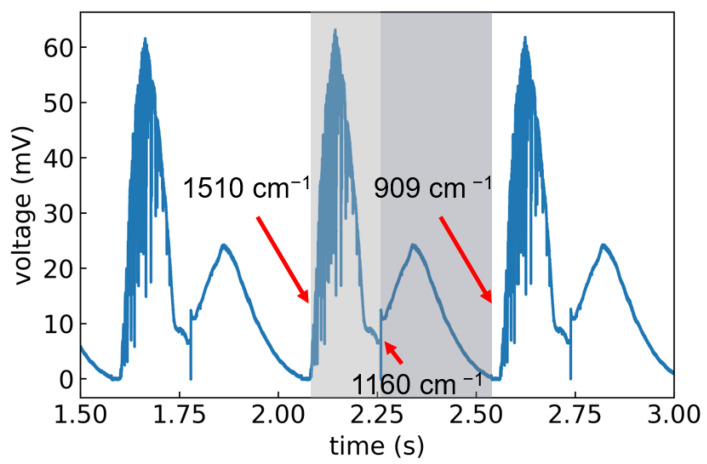
Voltage vs. time for three consecutive, continuous scans across the wavenumber range of 1510 to 909 cm^−1^. One scan is highlighted in grey and the start, stop and module switch positions are indicated.

Two tunable external-cavity quantum cascade laser (QCL) modules combined in a single laser system (MIRcat-Qt, Daylight Solutions GmbH, San Diego, CA, USA) were used. The MIRcat-Qt was operated at room temperature and emitted laser pulses with a length of 100 ns at a repetition rate of 1 kHz, in the wavelength range between 1510.5 and 909.1 cm^−1^. The emitted light had a linear (vertical) polarization. For most measurements, the laser was operated at a tuning speed of 10 μm s^−1^ which corresponded to 1397 cm^−1^s^−1^s and resulted in the acquisition of 2 spectra per second. The average output power of this particular system depended strongly on the chosen wavelength from 4.8 mW at 909 cm^−1^ to 47 mW at 1333 cm^−1^. Automatic switching between the two laser modules occurred at a wavelength of 1160 cm^−1^. In scan mode, as soon as the highest achievable wavelength of the set tuning range was reached, the grating jumped back to the starting point. The exemplary raw data acquired using a 4-stage Peltier-cooled MCT detector (IRDM-DCA) from NeoplasControl GmbH, Germany is shown in Figure 2. The curve contains three consecutive scans. One scan is highlighted in grey (module 1 in light grey and module 2 in dark grey) for clarity.

A small percentage (≈ 1%) of the laser light was reflected using a wedged ZnSe window (WW71050-E3,Thorlabs GmbH, Bergkirchen, Germany) and detected by an identical MCT detector as a power reference (Figure 1).The majority (≈99%) of the laser light was guided through a telescope to collimate the beam and adjust the size to a diameter of roughly 2 mm. The laser beam was then directed onto the sample using a 6 mm diameter gold mirror, which was positioned collinearly in front of the planoconvex-focusing ZnSe lens, which had a 3 inch diameter and focal length of 150 mm (Eksma Optics). It collected the backscattered and reflected sample signal and focused it onto the signal MCT detector. In accordance with the Gaussian’s lens formula, the image focal plane varied with detection distance. To account for this, the detector was placed on a linear translation stage (V-408 PIMag Linear Stage, Physik-Instrumente (PI) GmbH & Co. KG, Karlsruhe, Germany). A LiDAR system (DAN-30-150, Welotec GmbH, Laer, Germany) monitored the sample distance and subsequently moved the detector to the focal plane by a pre-defined look-up calibration. To be able to evaluate the influence of the sample position angle, a motorized sample stage was used to allow the automatic rotation of the sample by an angle α. For data acquisition, we used the 600 MHz UHF lock-in amplifier from Zurich Instruments AG, Switzerland. Both signal and reference channel were acquired by means of two built-in boxcar integrators. To reduce baseline fluctuations, a second averaging window was set and used for a differential measurement as background. The MCT detectors only reported a voltage at each point in time as can be seen in Figure 2. Thus, the signal needed to be assigned to the corresponding wavelengths. To that end, the wavelength and scan trigger signal that were provided by the laser were also recorded by the auxiliary inputs of the lock-in amplifier. The scan trigger indicated whether the laser was emitting light, whereas the wavelength trigger emitted a single digital pulse within a specified wavelength band and at a specific interval. The information from the position of the trigger signals was used later in the data analysis to assign a wavelength to the time traces. For the following characterization measurements, the alignment of the laser beam was kept fixed and the sample position was optimized, whereas for the application a beam-steering mirror was used for tracking purposes.

### 2.2. Sample Preparation and Measurement Procedure

**Figure 3:**
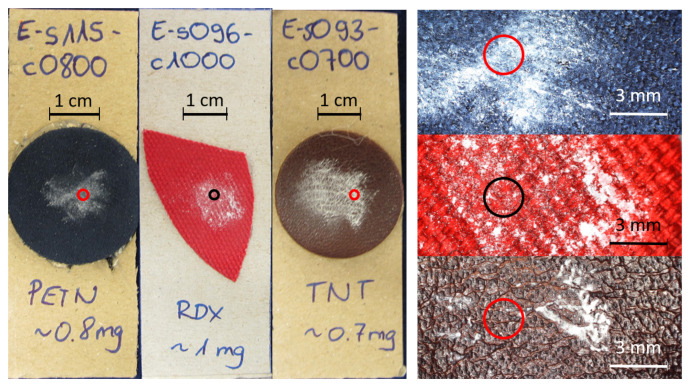
**Left**: Image of three different explosives samples on blue leatherette, red fabric and brown leather. **Right**: Corresponding microscopy images of the same samples. The red and black circles in all images represent the size of the laser beam.

To represent realistic samples, small amounts of explosives were deposited on various material: leather, synthetic fiber and jeans. Between 400 μg and 10 mg of each substance was gently rubbed or pressed onto the respective background material. Table 1 shows the different substances and materials that were prepared. Each substance (explosive or harmless) was prepared on each kind of material in two different amounts, resulting in 132 samples. An image of three exemplars is shown in Figure 3. Circles denote the beam diameter on the sample.

As published by Suter et al. [23] for coated surfaces [23], it appears that the sample distance only influenced the signal intensity while the angle α, at which the sample was placed with respect to the incoming laser beam, strongly influenced the spectral shape. Therefore, most measurements were performed at a fixed distance of 107 cm from the collecting lens and at 6 different angles of incidence (α = 0, 3, 6, 9, 12, 15${}^{{}^{\circ}}$). Each sample was measured at two different positions and each measurement comprised at least 5 scans, which yielded 60 measurements per sample and a total of 7920 spectra (Table 1). Here, we focus on a selection of samples and spectra that represent the general results of the data analysis. For the data reduction, e.g., the principal component analysis, all acquired data were considered. The full spectral analysis of the data will be discussed in a separate work.

Printed samples obtained from Fraunhofer ICT [24] having 50, 100, 250 or 1000 μg of RDX deposited on 1 cm^2^ area on aluminum and quartz substrates were measured as reference samples. Further measurements were performed with the ATR-FTIR spectrometer IRAffinity-1S from Shimadzu Deutschland GmbH, Germany. Each ATR-FTIR spectrum had 40 scans at a resolution of 2 cm^−1^.

### 2.3. Data Processing for Wavelength Calibration

**Figure 4:**
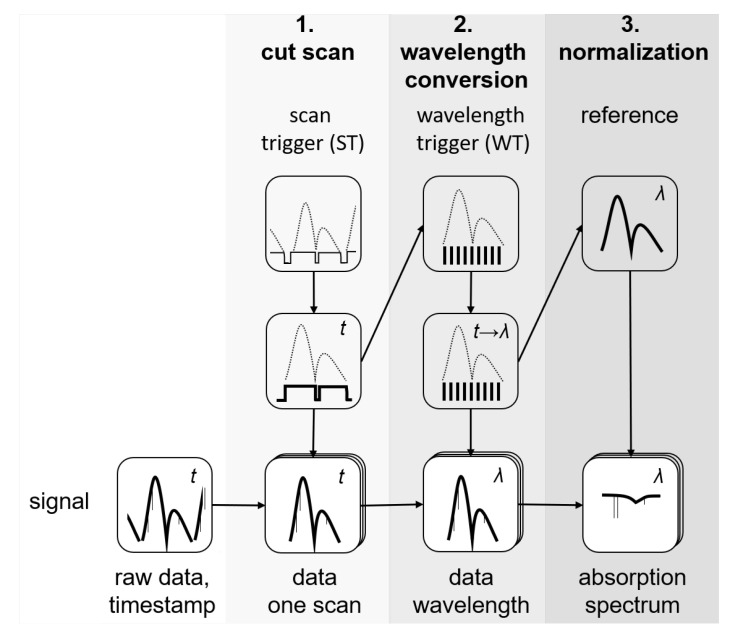
Flow chart of the data processing procedure.

The wavelength calibration followed an approach different from that in [25]. The data from the scan and wavelength triggers were used to calibrate the signal wavelength. This was done in multiple steps as shown schematically in the flow chart in Figure 4. In the first step (cut scan) the scan trigger signal was used to separate single scans and cut out time stamps that had no signal, i.e., when the laser was not emitting. In the second step (wavelength conversion) the wavelength trigger was used to assign the corresponding wavelength to the time stamps. Since these were identical for all channels, this procedure was applied to both the reference and the signal. Finally, the reference spectrum was used to normalize the sample spectrum (normalization).

## 3. Results and Discussion

### 3.1. Substance Detection

**Figure 5:**
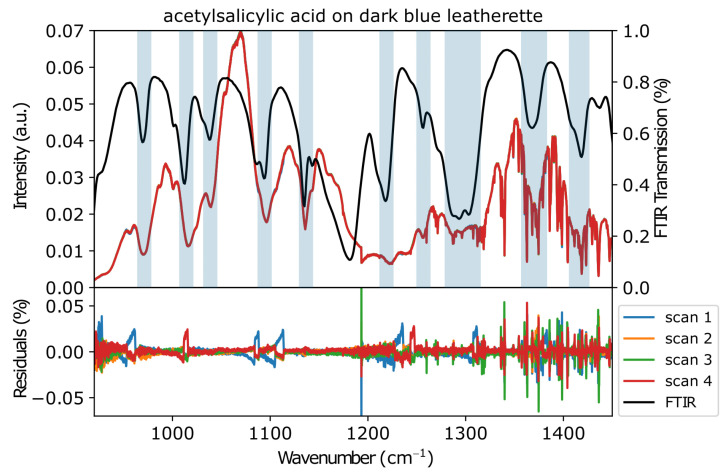
Consecutively acquired MIR spectra of ASA on dark-blue leatherette together with an FTIR spectrum (black). The light-blue lines are a guide to the eye. The bottom graph shows the residuals of the four measurements.

Figure 5 shows the spectra of acetylsalicylic acid (ASA) acquired with the new MIR setup. As can be seen from the residual plot at the bottom of the figure, the consecutively acquired scans have small variations. The largest appears at the position of the module switch at 1160 cm^−1^. Another relatively large variation between the scans arises from water vapor bands between 1300 and 1450 cm^−1^. The remaining variations amounted to a maximum of ±3%, which demonstrated the stability of the setup. The ATR-FTIR spectrum of ASA is shown in black and the most important peak positions are shaded light blue. The signals in both spectra overlapped to a great extent, proving that the sample was indeed being detected. Some differences in peak positions and shapes were to be expected due to the different spectra recording methods (scattered reflectance vs. ATR-FTIR) [26].

**Figure 6:**
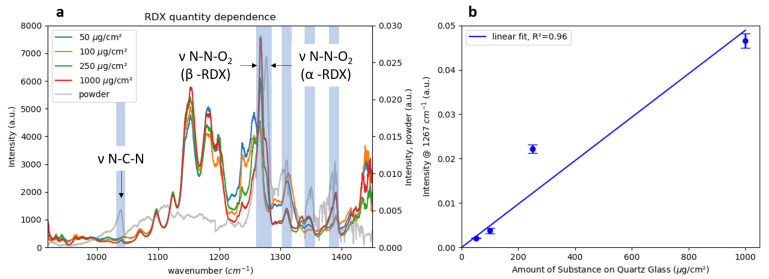
(**a**) MIR Spectra of 50, 100, 250 and 1000 μg of RDX printed on 1 cm^2^ of a quartz substrate shown with the spectrum of bulk RDX powder. The main peaks are labeled according to the peak assignment of Figueroa-Navedo et al. [27]. (**b**) Maximum signal intensity at 1267 cm^−1^ as a function of the amount of RDX on the quartz substrate. Each data point is the average of three measurements at different positions of the sample. The error bars represent the standard deviation.

To investigate the setup performance, the spectra of samples with varying amounts of RDX deposited on quartz glass were measured at a distance of 90 cm. The spectra shown in Figure 6a were normalized by a bare quartz reference spectrum and divided by their respective maxima to increase the comparability since the signal strength varied greatly between measurements of different amounts. For comparison, a spectrum of pure RDX powder is shown in grey. A comparison of the peak positions clearly showed that RDX signatures over the whole spectral range were present in all the MIR data. The main vibrational signatures were assigned to νN-C-N ( 1040 cm^−1^), νN-N-O${}_{2}$ stretch in β-RDX ( 1266 cm^−1^) and νN-N-O${}_{2}$ stretch in α-RDX ( 1277 cm^−1^) [27]. Additionally, a number of signals that could not be related to RDX were visible in the spectra of the printed samples, especially between 1100 and 1200 cm^−1^. The most prominent RDX spectral feature around 1260 cm^−1^ was present in all spectra, while the second-most intense signal at roughly 1040 cm^−1^ could not be observed in the spectra of the samples containing only 50 and 100 μg RDX. The signal intensities were higher at 1260 cm^−1^, which was close to the maximum in laser power at roughly 1330 cm^−1^. At 1000 cm^−1^, the laser output was significantly lower (see Figure 2), which resulted in a decreased signal-to-noise ratio in that region. The differences in peak positions between the spectra of the printed samples and the powder sample between 1250 and 1350 cm^−1^ were attributed to the presence of different crystal structures of α-RDX and β-RDX, which are known to show differences in their infrared spectra [27,28]. To allow for the correct classification in special cases like this, the spectra of both crystal structures had to be included in the classification model.

To test the linearity of the detection system, Figure 6b shows a maximum signal intensity at 1276 cm^−1^ for the deposited amount of RDX and a linear fit with an $R^{2}=0.96$. It should be noted that the amounts were not equal to the detected amounts, which were illuminated by the laser and visible to the detection system. The circular beam had a diameter of 2 mm on the sample which resulted in an illuminated area of 0.03 cm${}^{2}$. Assuming a homogeneous deposition of RDX on the glass substrates over the whole covered area of 1 cm${}^{2}$, the detected amounts of RDX are given in Table 2.

### 3.2. Detection on Fabrics and Influences on Spectral Shape

**Figure 7:**
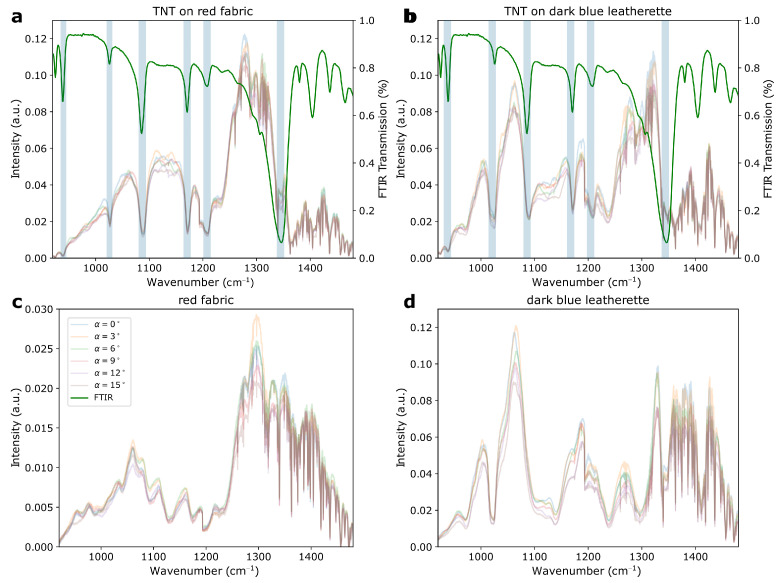
(**a**) TNT on red fabric and (**b**) TNT on dark-blue leatherette at various angles of incidence. The green spectrum presented in both graphs is the FTIR spectrum of TNT. The light-blue stripes are a guide for the eye and mark the positions of the signals. (**c**,**d**) represent the spectra of the bare red fabric and dark-blue leatherette at various angles of incidence, respectively. The legend shown in (**c**) is valid for all four graphs.

The measurements of explosives on various background fabrics and at a variety of angles of incidence revealed a number of influencing factors that changed the spectra in different ways. The first was the reflectivity of the background material, which was observed when comparing the ASA spectra in Figure 5 with the RDX spectra in Figure 6a. While the former showed dips at the position of the ATR–FTIR spectral signature dips, the latter exhibited peaks at the positions of the ATR–FTIR dips. According to Ref. [29], this discrepancy arose from the varying reflectivity of the background material and posed a challenge to automatic data analysis. The influence of the background material was also visible in the exemplary TNT spectra on red fabric and dark-blue leatherette presented in Figure 7a,b, respectively. The spectra of the bare background materials are shown in Figure 7c,d for comparison.

The spectra of the background materials showed significant deviations from the spectral envelope of the laser. However, after comparing Figure 7a with Figure 7c and Figure 7b with Figure 7d it was clear that the additional, unique spectral features that arose from TNT were only present in Figure 7a,b. Upon comparing both spectra of TNT it was apparent that the background material had a strong influence on the overall spectral shape, e.g., between 1000 and 1200 cm^−1^, where the the baseline showed pronounced differences. Additional spectral variations in the sample spectra appeared because of the different angles under which the spectra had been measured. All the slightly transparent spectra in Figure 7a,b were acquired at various angles of incidence α between 0 and 15${}^{{}^{\circ}}$. Here, the influences were expressed as fluctuations in the baseline of the spectra: for example, between 1100 and 1200 cm^−1^. This angle dependency was the result of a combination of effects, including hitting different positions when turning the sample and changing the laser spot sizes due to distortion of the circular beam profile at higher incidence angles. The influence of the incidence angle on the spectra was clearly visible, but less dominant than the influence from the underlying background material.

### 3.3. Normalization of Spectra

**Figure 8:**
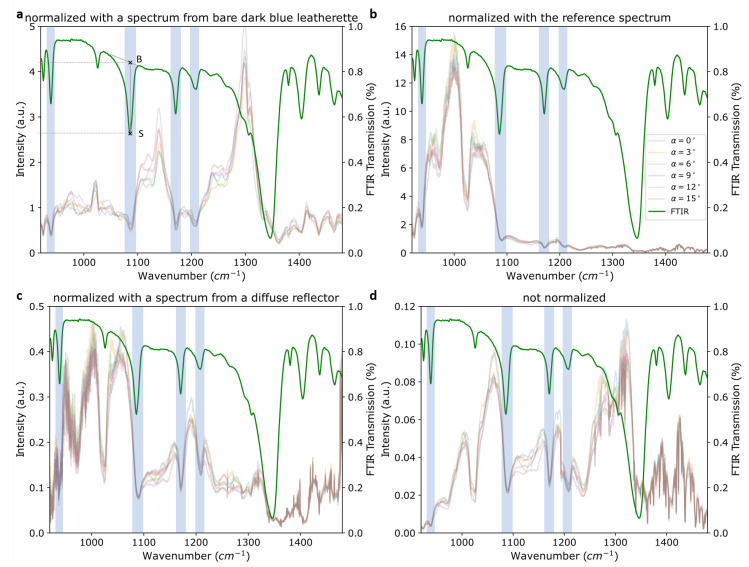
Spectra of TNT on dark-blue leatherette at various angles of incidence normalized with (**a**) the spectrum from bare dark-blue leatherette, (**b**) the spectrum acquired simultaneously with the reference detector and (**c**) the spectrum from a diffuse silver-coated reflector. (**d**) shows the raw, i.e, non-normalized, spectrum of TNT on dark-blue leatherette. The green spectrum shown in all four graphs is the ATR–FTIR spectrum of TNT powder. The legend, which is only shown in (**b**), is valid for all graphs in this figure. The peaks used to evaluate the normalization are shaded blue. An example of the points used for calculation of the baseline-to-signal ratios is indicated in the FTIR spectrum in (**a**). B and S are the points used for the baseline and the signal intensity, respectively.

As discussed in the previous section, various factors not related to the sample substance itself influenced the spectral shape. Two main examples were the laser intensity and background material. As these factors may have clouded the spectral information we were looking for, it was beneficial to minimize their impact by normalizing the spectra. For the acquired data three approaches were tested and their results are presented in Figure 8.

To evaluate the results, the intensity of the spectral baseline (B) divided by the intensity of the sample signal (S) was calculated for four TNT peaks (940, 1090, 1180 and 1210 cm^−1^) for each method. Comparing these “baseline-to-signal” ratios was a quantitative measure for signal contrast achieved by the respective normalization method. The signals are highlighted in Figure 8 and the intensity values were estimated according to the example sketched in Figure 8a. For clarity, the FTIR spectrum was used. The results for all three normalization methods are listed in Table 3. For absorption dips, as in the data in Figure 8, the resulting ratios were >1, and for peaks they were <1. A higher baseline-to-signal (B–S) ratio indicated increased visibility of the peak and thus a better correction. Generally, the best results were achieved by using a diffuse reflector as presented in Section 3.3.3. Further details will be discussed in the respective subsections. In our measurements, the spectra from two different points on the same sample with roughly the same amount of substance showed variations comparable to the ones because of changes in illumination angle, as discussed in Section 3.2. Accordingly, normalization results were also very similar for different spots on the sample.

#### 3.3.1. Normalization by Background Material

To minimize influence from the background material on the sample spectrum, the spectra ($S_{TNT}$) were normalized with the spectra from the respective background material ($S_{BM}$) as described in Equation (1).

(1) $$S_{TNT}^{norm}\left(\lambda \right)=\frac{S_{TNT}\left(\lambda \right)}{S_{BM}\left(\lambda \right)}$$

The resulting normalized spectrum of TNT on dark-blue leatherette is shown in Figure 8a. However, this normalization procedure did not yield a significant improvement in the clarity of these spectra as can be seen when comparing the normalized signals in Figure 8a with the non-normalized spectra in Figure 8d. The TNT signal at 1090 cm^−1^, for example, was significantly less pronounced after normalization as the B–S ratio decreased from 2.73 to 2.03. This was explained by the changing contribution of the background material when covered by TNT particles.

#### 3.3.2. Normalization by Reference Detector

Figure 8b shows the TNT spectra normalized by the signal acquired simultaneously with the reference detector ($S_{Ref}$) according to Equation (2).

(2) $$S_{TNT}^{norm}\left(\lambda \right)=\frac{S_{TNT}\left(\lambda \right)}{S_{Ref}\left(\lambda \right)}$$

This results in a large spectral distortion as the laser passes different optics on the way to the sample compared to the rather direct path to the reference detector (see Figure 1). However, the calculated B–S ratios were all comparable to the non-normalized spectrum, indicating that this distortion did not influence the peak visibility but only the total intensity across the spectrum. Another influence that may have interfered with this analysis procedure is the mutual nonlinearity of the two MCT detector responses. To remove those factors, a quick approach for their calibration was tested by placing neutral density (ND) filters of varying optical thicknesses in the beam path in front of the MCT detectors. However, the resulting spectra acquired by both detectors were heavily clouded by interference fringes caused by the ND filters. Since this type of detector calibration with ND filters did not succeed, a normalization using the reference detector signal did not appear feasible in the current configuration.

#### 3.3.3. Normalization by Diffuse Reflector

In a different approach, a diffuse reflector with a silver coating was placed at the sample position and the resulting spectrum was used for normalization. To account for variations in laser power, the reference that was acquired simultaneously had to be taken into account as well. The normalization process resulting in the signal $S_{TNT}^{norm}$ of the spectra in Figure 8c is depicted in Equation (3). Here, $S_{TNT}$ is the original TNT signal and $S_{diff}$ is the signal from the diffuse reflector. $S_{TNT}^{ref}$ and $S_{diff}^{ref}$ are the signals from the reference detector acquired simultaneously to the TNT and diffuse reflector signals, respectively.

(3) $$S_{TNT}^{norm}\left(\lambda \right)=\frac{S_{TNT}\left(\lambda \right)}{S_{TNT}^{ref}\left(\lambda \right)}\times \frac{S_{diff}^{ref}\left(\lambda \right)}{S_{diff}\left(\lambda \right)}$$

Normalization using the diffuse reflector produced a significant improvement in signal visibility compared to the two previously discussed methods. For example, the two signals at 1180 and 1210 cm^−1^ were substantially more pronounced in Figure 8c than in Figure 8a,b. The calculated B–S ratios were significantly higher for this method than for the previous ones, except for the peak at 1210 cm^−1^. They were also moderately higher than in the non-normalized spectrum in panel Figure 8d, indicating the usefulness of this normalization method.

### 3.4. Removal of Atmospheric Influences

**Figure 9:**
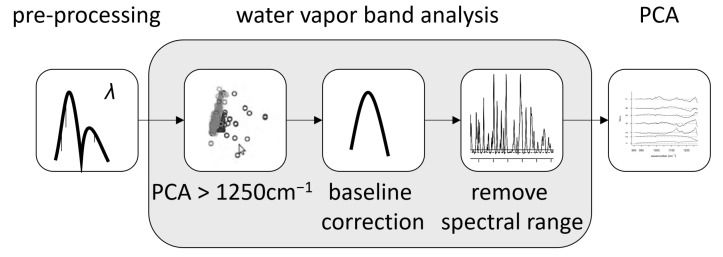
Flow chart of the water band analysis.

To assess whether it was possible to distinguish the spectral features automatically, a principal component analysis (PCA) was performed that included all measured data. It showed that a significant contribution to the first components were the water vapor absorption lines that overshadowed additional variations in the spectra. It was thus necessary to remove these water vapor bands for analysis. For free space (standoff) applications, absorption by atmospheric water could not be avoided experimentally, and needed to be removed during post-processing. For that purpose, a PCA at hlwavenumbers above 1250 cm^−1^ was performed. Here, the first component mainly included the water bands as well as the broad intensity curve from the laser. The broad part was removed using a baseline correction (asymmetric least squares, λ=3,p=0.01). In this way, only the water vapor absorption bands remained. After choosing an appropriate signal threshold of $4\times 10^{-4}$ V, the intense water bands were mostly removed from the original spectrum without losing large spectral sections. The flow chart in Figure 9 shows the step-wise process of this analysis.

**Figure 10:**
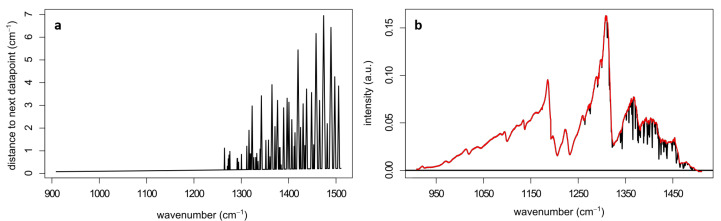
(**a**) Amount of data points (*y*-axis) being removed from the spectra during the water band analysis at each wave number and (**b**) a spectrum of ASA (red) where the water bands were removed from the spectrum together with the removed water bands shown in black. The spectrum shown here was acquired using the wavelength scan mode of the laser, while all other spectra were acquired using the wave number scan mode, which resulted in a higher accuracy in the *x*-axis calibration.

The largest gap in data points that results from this water band analysis amounts to 7 cm^−1^. Figure 10a shows the amount of removed data points at each wave number. Since we were dealing with condensed phase spectra, the minimal width of infrared signals of the samples was expected to be on the order of 10 cm^−1^. To show that the sample signals were indeed still visible after the water band analysis, a spectrum of ASA after the procedure is presented by the red spectrum in Figure 10b. The removed parts of the spectrum are shown in black. This pre-processing step removed between 10 and 15% of the total variance of the raw data matrix depending on the overall signal variation.

### 3.5. PCA for Measured Substances

**Figure 11:**
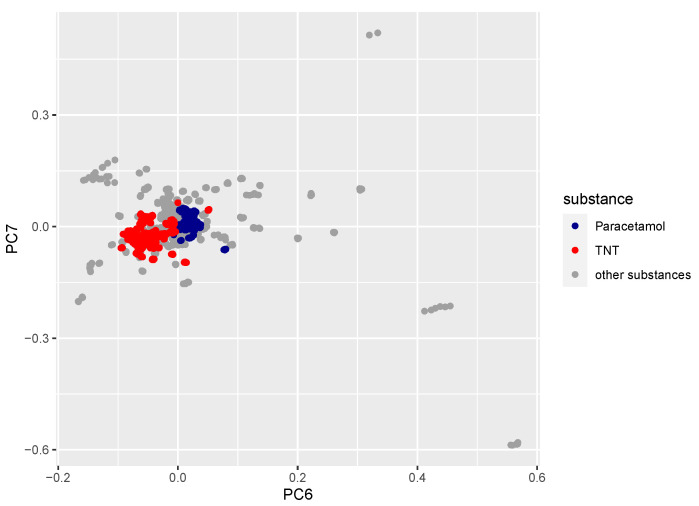
Loading of component 7 plotted vs. loading of component 6 for paracetamol (blue), TNT (red) and all other substances (grey) included in the measurement campaign (Table 1). Approximately 800–900 data points were used per substance, i.e., every scan of every sample of TNT/paracetamol on all different background materials and all sample spots and all different illumination angles were measured.

The loadings from the PCA performed after the water vapor band removal still had some influence from the remaining bands; however, the main contribution was removed. The dominating principal components 1 and 2 represented variance due to the physical and instrumental sources, e.g., laser intensity fluctuations, so they could not be used to discriminate the samples. Relevant chemical variance was contained in principal components 3–7 only, which described about 1 % of the total variance. In Figure 11 the loadings of principal component 7 are plotted against the loadings of component 6, representing about 0.1 % of the total variance. There was a clear separation between paracetamol (blue) and TNT (red), a harmless substance and an explosive. However, this comparison—based on principal components 6 and 7 only—did not allow for the separation of all measured substances. The sum of all other substances (grey) overlapped in the plot shown here. The other principal components (3–5) showed a very moderate or no separation between TNT and paracetamol; therefore, they were not shown here. To be able to distinguish among all substances, improvements to the analysis procedure of all principal components and significantly more measurements will be needed.

## 4. Summary and Outlook

We showed that the newly developed setup enabled the detection of trace (μg) amounts of explosives at a distance of roughly 1 m. The measurements were performed very fast: one full scan took only half a second.

Furthermore, successful measurements of these trace amounts on a number of realistic samples at various angles of incidence were presented. An approach for data pre-treatment with the removal of the spectral regions dominated by water vapor absorption lines was proposed and tested successfully. We used a wavelength calibration method that was accurate enough to reliably reproduce the removal of water vapor bands from the spectra. The water vapor analysis reduced the overall data variance by up to 15% and is a promising first step toward the automatic assignment of spectra to the corresponding explosive samples.

Three different methods for normalizing the spectra were tested and evaluated. Of these three methods, only normalization with a diffuse reflector spectrum showed significant improvement in signal visibility. However, the method needs to be improved to eliminate the influence of factors unrelated to the sample substance. In addition, a substantially larger set of independent data containing around 100 samples per explosive will be required. This is necessary to achieve reasonable precision [30] in the internal validation of the classification model. For that purpose, automation of the measurement procedure will be implemented.

To increase the investigated area and thus the chances of detecting an explosive trace on a surface, two options are possible: first, expanding the laser beam to cover a larger area, possibly combined with scanning the surface. Expanding the beam diameter would require a laser system with increased power to maintain the intensity on the illuminated surface. Thus, ensuring the desired sensitivity in an acceptable detection time. Second, pre-screening the surface using imaging methods to identify hotspots or regions of interest.

## Figures and Tables

**Table 1:** List of measured background materials and substances.

Background Materials (BGM)	Explosives (E)	Harmless Substances (HS)
light-blue jeans	RDX	acetylsalicylic acid
dark-blue jeans	TNT	cinnamic acid
red canvas	PETN	paracetamol
black synthetic fiber	ammonium nitrate	sucrose
brown leather		ascorbic acid
dark-blue leatherette		malic acid
		citric acid
2 × (6 Background Materials × (4 Explosives + 7 Harmless Agents)) = 132 Samples		
2 Positions × 6 Angles × 5 Scans = 60 Measurements		
132 Samples × 60 Measurements = 7920 Spectra		

**Table 2:** Deposited and detected amount of RDX assuming homogeneous deposition.

Total Amount of RDX (μg)	Detected Amount (μg)
50	1.5
100	3.0
250	7.5
1000	30

**Table 3:** Calculated baseline-to-signal ratios for the exemplary spectra in Figure 8.

	Signal Position			
Method (Section)	940/cm	1090/cm	1180/cm	1210/cm
Background (Section 3.3.1)	2.10	2.03	1.80	1.56
Reference (Section 3.3.2)	2.26	2.88	1.98	1.47
Diff. reflector (Section 3.3.3)	2.72	2.96	2.10	1.51
Not normalized	2.25	2.73	2.04	1.42

## Data Availability

The data presented in this study are available on request from the corresponding author. The data are not publicly available as this work is part of an ongoing project.
